# Neutralising antibody response in domestic cats immunised with a commercial feline immunodeficiency virus (FIV) vaccine

**DOI:** 10.1016/j.vaccine.2015.01.028

**Published:** 2015-02-18

**Authors:** Paweł M. Bęczkowski, Matthew Harris, Navapon Techakriengkrai, Julia A. Beatty, Brian J. Willett, Margaret J. Hosie

**Affiliations:** a Centre for Virus Research, University of Glasgow, Glasgow, United Kingdom; b Small Animal Hospital, University of Glasgow, Glasgow, United Kingdom; c Valentine Charlton Cat Centre, University of Sydney, Sydney, NSW, Australia

**Keywords:** FIV vaccine, Vaccine induced protection, Neutralising antibodies, FIV, feline immunodeficiency virus, FeLV, feline leukaemia virus, NAb, neutralising antibody, RT, reverse transcriptase, GARD, genetic algorithm recombination detection, ML, maximum likelihood, NJ, neighbour joining, AIC, akaike information criterion, bp, base pair

## Abstract

Across human and veterinary medicine, vaccines against only two retroviral infections have been brought to market successfully, the vaccines against feline leukaemia virus (FeLV) and feline immunodeficiency virus (FIV). FeLV vaccines have been a global success story, reducing virus prevalence in countries where uptake is high. In contrast, the more recent FIV vaccine was introduced in 2002 and the degree of protection afforded in the field remains to be established. However, given the similarities between FIV and HIV, field studies of FIV vaccine efficacy are likely to advise and inform the development of future approaches to HIV vaccination.

Here we assessed the neutralising antibody response induced by FIV vaccination against a panel of FIV isolates, by testing blood samples collected from client-owned vaccinated Australian cats. We examined the molecular and phenotypic properties of 24 *envs* isolated from one vaccinated cat that we speculated might have become infected following natural exposure to FIV. Cats vaccinated against FIV did not display broadly neutralising antibodies, suggesting that protection may not extend to some virulent recombinant strains of FIV circulating in Australia.

## Highlights

• FIV vaccinated cats screened for neutralising antibodies
• Homologous neutralisation in 50% of cats tested
• No heterologous neutralisation

## 1 Introduction

Throughout the history of retroviral vaccine development, only two vaccines have made it to market, the vaccines for the feline retroviruses FIV and FeLV. FeLV vaccines were introduced over thirty years ago and since then have made a significant impact upon the prevalence of infection [1]. The first FIV vaccine was introduced in the US in 2002. Fel-O-Vax FIV (Boehringer-Ingelheim) induces 80% protection against experimental [2] and contact challenge [3], with protection also extending to heterologous challenge [4]. Given the similarities between FIV infection of cats and HIV infection of humans, a broader understanding of the mechanisms of immunity to infection with FIV may inform the development of candidate HIV vaccines. An effective HIV vaccine has proved elusive [5] and the insights offered by the study of retroviral immunity in other species may direct future research efforts along a more fruitful pathway.

The success of FeLV vaccines may reflect the ability of a proportion of cats to recover from natural infection. In contrast with FeLV, spontaneous resolution of infection has been documented in neither HIV nor FIV infection [6,7], posing a major obstacle to lentiviral vaccine development. Numerous experimental HIV vaccine candidates have been developed, with outcomes ranging from complete protection to enhancement of infection [5]. Four HIV vaccines were advanced from testing in non-human primate models to phase IIb or III efficacy trials in human volunteers [8,9]. These included VaxGen gp120 (B/B′ and B/E) tested in trials in the USA [10,11] and Thailand [12], the Merck Ad5-HIV-1 tested in the STEP trial [13,14] and ALVAC + gp120 tested in the RV144 study [15]. The Merck vaccine trial was halted prematurely when it became evident that vaccination increased the risk of HIV acquisition [16], but most promising was the 30% protection observed in the RV144 study [17]. These contrasting findings raise the question: is our current understanding of lentiviral biology and immune correlates of protection sufficient to design a safe and fully efficacious lentiviral vaccine?

FIV provides a unique opportunity to conduct comparative studies to define the mechanisms of vaccine protection against lentivirus infection [18]. Several FIV vaccine candidates have been tested, yielding valuable insights into the virus biology and correlates of protection. To date, whole inactivated virus and fixed infected-cell vaccines have proved to be the most successful [18–20], leading to the commercial production of the whole inactivated virus, dual-subtype FIV vaccine [2]. Following safety and efficacy evaluation by USDA (US Department of Agriculture), the FIV vaccine was launched in the US in 2002 [21] and subsequently has been licensed for veterinary use in Canada (2003), Australia and New Zealand (2004) and Japan (2008).

Although a lack of protection against the pathogenic primary FIV isolate GL8 was evident experimentally [22], no independent research has been published evaluating the commercial vaccine's efficacy under field conditions [4]. Here we identified rare samples from client-owned cats that had been vaccinated against FIV, evaluating the breadth of neutralising antibodies (NAbs) induced following FIV vaccination.

## 2 Materials and methods

### 2.1 Blood samples

Samples used in this study were collected according to the University of Sydney Animal Ethics Committee approvals (N00/6-2009/1/4985). A search of the electronic medical records of the Valentine Charlton Cat Centre (VCCC), University of Sydney from January 2005 to September 2010 identified cats with a history of FIV vaccination. Informed owner consent was available (University of Sydney ethics approval number N00/6-2009/1/4985) to use blood samples from 8 vaccinates of known ELISA and FIV PCR status (Gribbles Veterinary Laboratories, Victoria, Australia) and a further 2 vaccinates tested negative using a quantitative PCR to detect FIV *gag* (data not shown). One vaccinated cat (SV1) tested positive for FIV proviral DNA (Table 1). Blood samples were collected into heparinised collection tubes. Samples were centrifuged at 2000 rpm (∼370 × *g*) for 10 min and plasma and cell fractions were separated and stored frozen at −80 °C.

### 2.2 Amplification and cloning of FIV env

The complete FIV *envs* from cat SV1 were amplified directly from whole blood using a nested PCR protocol (Table S1). First round PCR products were amplified using Phusion Blood Direct II Polymerase (Finnzymes, Thermo Fisher Scientific) and the nucleic acid sequence of the first-round PCR product informed primer design for the second round PCR, performed using High Fidelity Master (Roche). Strain-specific primers for the second round PCR reactions incorporated restriction sites to facilitate sub-cloning into the eukaryotic expression vector VR1012 [23]. Thus cloned *envs* were transformed into Escherichia Coli MAX Efficiency^®^ DH5α™ Competent Cells (Invitrogen). In total, 24 clonal *env* variants were obtained from cat SV1; however, since sequence analysis revealed that 12/24 amplicons contained only synonymous mutations, we proceeded to produce pseudotypes bearing the 12 Envs with unique amino acid sequences. Therefore these twelve FIV *env* expression constructs were co-transfected transiently with HIV pNL4-3-Luc-E^-^R^-^luc plasmid (an *env*-deleted HIV provirus containing a luciferase reporter gene) [24] into HEK 293T cells [25] using Superfect Transfection Reagent (Invitrogen). Following a 72 h incubation in 6 well culture clusters (Corning), culture fluids containing HIV(FIV)-luciferase pseudotypes (hereafter referred to as HIV(FIV)-luc, with the FIV Env in parenthesis) were harvested, centrifuged at 1000 rpm (∼200 × *g*) for 5 min, passed through 0.45 μm filters and stored at −80 °C until required. HIV(FIV) luciferase pseudotypes (*n* = 43) were prepared, bearing 12 Envs from cat SV1, 24 field Envs isolated from 24 naturally infected American cats [26] and 7 reference FIV Envs reported previously [27–33], in order to assess neutralising antibody (NAb) responses in plasma samples and to determine the nature of the Env-receptor interaction.

### 2.3 Neutralisation assays

Plasma samples from 10 vaccinated cats were tested for NAbs against a panel of 31 HIV(FIV)-luc pseudotypes bearing heterologous Env (Table 3). The exceptions were samples from SV6, SV3, and SV2, which were limiting and could only be tested against 6, 21 or 25 pseudotypes, respectively. Due to the high sequence homology observed, pseudotypes bearing 12 SV1 Envs, representative of the 24 Envs cloned, were tested additionally for sensitivity to autologous neutralisation.

Tenfold dilutions of each plasma sample were prepared in complete RPMI 1640 medium (Invitrogen), from a starting dilution of 1 in 10. Next, 25 μl of each plasma dilution (1 in 10, 1 in 100 and 1 in 1000) were incubated in triplicate for 1 h at 37 °C with 25 μl of HIV(FIV)-luc pseudotype before 5 × 10^4^ of CLL-CD134 cells [34] were added in 50 μl. Following a 72 h incubation in CulturPlate™-96 assay plates (Perkin Elmer), luciferase activity was quantified by the addition of 100 μl of Steadylite HTS™ (Perkin Elmer) substrate and single photon counting, using a MicroBeta luminometer (Perkin Elmer). Fold neutralisation was calculated by dividing the mean luciferase counts of control wells containing no plasma (NP luc) with the mean luciferase counts for wells containing 1 in 10 plasma dilutions (P luc). Plasma samples were classified according to neutralisation potency, using the cut-off values shown in Table 2.

### 2.4 Assaying receptor utilisation

Feline cells expressing feline CD134 (MCC FFF), a chimaeric human × feline CD134 (MCC FFH) or human CD134 (MCC HHH) [34], and a canine cell line modified to express feline CD134 (CLL-CD134) [34], were seeded at 1 × 10^4^ cells per well in triplicate in a CulturPlate™-96 assay plate (Perkin Elmer). The cells were infected with 50 μl of each HIV (SV1)-luc pseudotype, alongside reference controls of HIV (GL8)-luc and HIV (B2542)-luc. After incubation for 72 h at 37 °C in an atmosphere of 5% CO_2_, the luciferase activity was quantified as described above.

### 2.5 Sequences and phylogenetic analyses

Twenty-four VR1012 plasmids expressing SV1 FIV *envs* were sequenced using the Big Dye Terminator v1.1 kit. The full length FIV *env* sequence (approx. 2500 bp) from each clone was assembled using 4 sequencing reads overlapping by approximately 200 bp and manually checked for mismatches. Nucleotide and peptide sequence alignment was performed using the Muscle algorithm [35] in MEGA5 [36]. Evolutionary divergence between sequences was calculated using the Maximum Composite Likelihood model [37]. A phylogenetic tree comprising the complete *env* sequences was constructed using the maximum likelihood (ML) method under HKY nucleotide substitution model [36] in MEGA5. Sequences were analysed using the Datamonkey webserver [38], employing the genetic algorithm recombination detection (GARD) method [39]. Neighbour joining (NJ) trees for each recombination segment (identified by GARD and assessed by Akaike Information Criterion (AIC) [40]) were prepared for presentation in FigTree v 1.3.1 (http://tree.bio.ed.ac.uk/). A representative figure visualizing recombination breakpoints was generated in SimPlot v 3.5.1 [41]. Highlighter analysis was performed using the highlighter tool available at the Los Alamos National Laboratory server (www.hiv.lanl.gov). Graphs were created in GraphPad Prism v 5.00 (GraphPad Software).

## 3 Results

### 3.1 Breadth of the neutralizing antibody response in vaccinated cats

To assess the breadth and strength of NAbs in cats vaccinated with the Fel-O-Vax FIV vaccine, 10 plasma samples collected from vaccinated field cats were tested for neutralisation against a panel of pseudotypes bearing a range of FIV Envs, including Envs from reference subtype A, B and C isolates and primary field isolates of FIV (Table 3). Plasma samples from ten vaccinated cats displayed variable neutralisation of the pseudotypes but plasma SV5 strongly neutralised five pseudotypes bearing Envs of US field isolates, SV4 strongly neutralised four pseudotypes, one bearing the Env designated KKS and a further three bearing US field isolate Envs and SV1 strongly neutralised three pseudotypes bearing Envs of US field isolates. The pseudotype bearing the Env designated KKS (clade A) was closely related to FIV Petaluma Env (one of the isolates within the FIV vaccine) and was neutralised by nine of the ten plasma samples tested. Three pseudotypes bearing Envs cloned from naturally infected US cats (P14, clade A/B; M49, clade B; and P6, clade B) were strongly neutralised by five, three and two plasma samples, respectively (Table 3).

### 3.2 Vaccinated, provirus positive cat SV1: Phylogenetic inference

**Fig. 1:**
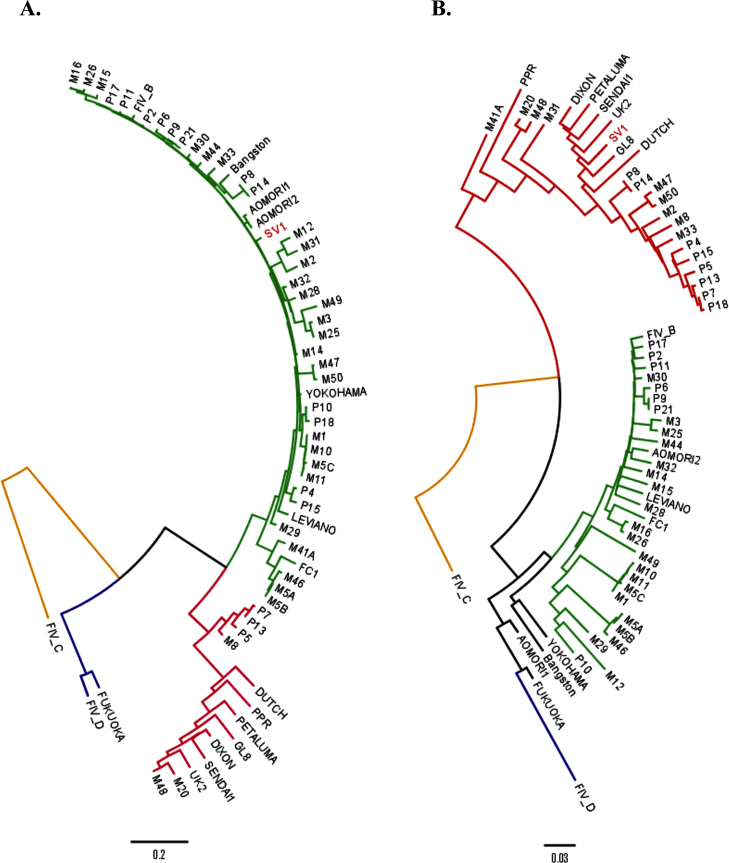
Neighbour joining trees for each of two GARD determined segments of representative SV1 sequence; tree (A) represents phylogenetic inference of the first segment of the *env* (1–483 bp) and tree (B) of the second segment (484–2562 bp). Both trees are based on: (1) one sequence representative of 24 *env* genes from cat SV1 (red tip), (2) 43 entire *env* sequences from cats naturally infected with FIV in the US [26], from which the Envs were used to prepare pseudotypes for neutralisation studies in the present study and (3) 17 full length *env* sequences derived from GenBank; Aomori 1 [D37816], Aomori 2 [D37817.1], FIV C [AF474246.1], Dixon [L00608.1], Dutch [X60725], Fukuoka [D37815.1], Sendai 1 [D37813.1], Shizuoka [D37811.1], UK2 [X69494.1], UK8 [X69496.1], USIL2489 [U11820.1], Yokohama [D37812.1], Petaluma [M25381.1], PPR [M36968.1], Leviano [FJ374696.1], Bangston [AY620002.1] and FC1 [AY621093.1]. The first segment of the SV1 *env* sequence (red tip) clusters with clade B isolates (green nodes), while the second segment (red tip) is more closely related to the clade A isolates (red nodes) that include GL8. Trees are rooted on the FIV clade C reference *env* sequence and are drawn to scale with branch lengths denoting the number of substitutions per site. (For interpretation of the references to color in this figure legend, the reader is referred to the web version of this article.)

Twenty-four *env* sequences cloned from cat SV1 were identical, or near identical, with an overall mean intra-host diversity of 0.1% (Fig. S3). Maximum likelihood analysis revealed that cat SV1 harboured viruses containing clade A *envs* (Fig. S4). However, following rigorous recombination testing, it was evident that all *envs* from cat SV1 were clade A/B recombinants. GARD analysis indicated one breakpoint with significant topological incongruence (*p* = 0.00120) at position 483 of the nucleotide sequence alignment. Thus the first segment of GARD spliced *env* was assigned to Clade B while the remaining fragment clustered together with clade A and was relatively closely related to the GL8 strain of FIV (K2P distance of 7%), (Fig. 1)*.*

### 3.3 Autologous neutralising antibody response

**Fig. 2:**
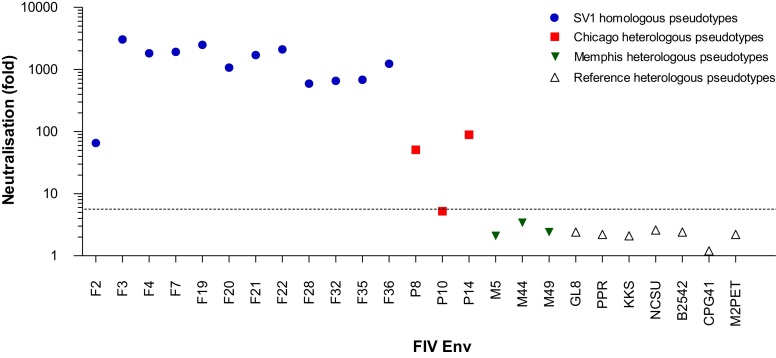
Neutralisation of plasma SV1 against HIV(FIV)luc pseudotypes bearing: (1) 12 autologous SV1 Envs (blue circles), (2) 3 heterologous Envs representative of Memphis field isolate Envs (red squares), (3) 3 heterologous Envs representative of Chicago field isolate Envs (green triangles) and (4) 7 heterologous Envs of reference FIV isolates (white triangles). Fold neutralisation was calculated by dividing the mean luciferase counts of control wells containing no plasma with the mean luciferase counts for wells containing 1 in 10 plasma dilutions. All pseudotypes bearing autologous Envs, but only three bearing heterologous Envs, were strongly neutralised by plasma SV1. For clarity, only 6 representative heterologous Envs from the US cats (Chicago and Memphis) are included; the complete neutralisation data is shown in Table 3. The dashed line indicates 5.6 fold neutralisation; plasma samples displaying neutralisation greater than 5.6 fold were considered to be ‘strongly neutralising’. (For interpretation of the references to color in this figure legend, the reader is referred to the web version of this article.)

Plasma SV1 was one of three samples that displayed the broadest heterologous neutralisation (Table 3). Compared to the moderate heterologous neutralisation observed, SV1 strongly neutralised all pseudotypes bearing autologous Envs (ranging from 65 to 3042-fold neutralisation, Fig. 2).

### 3.4 Receptor utilisation

**Fig. 3:**
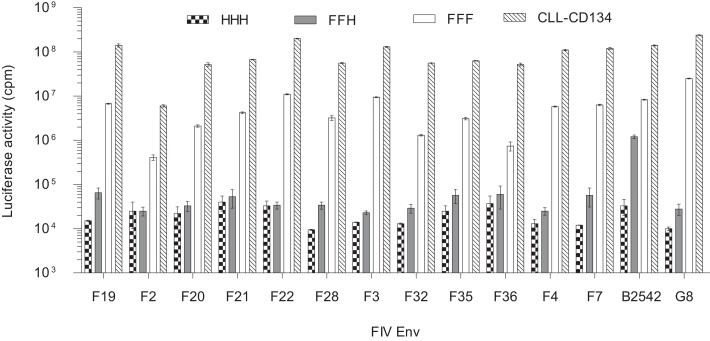
A panel of cell lines bearing chimaeric human × feline CD134 molecules was used to assess receptor usage by 12 pseudotypes bearing Envs from cat SV1. CD134 consists of 3 CRDs; HHH represents MCC cells expressing the entire human CD134, while CLL-CD134 represents cells expressing the entire feline CD134. These constructs served as negative and positive controls, respectively. FFF represents MCC cells expressing feline CD134 while FFH represents MCC cells expressing a chimeric feline/human CD134 with the CRD2 domain comprising the human sequence. These cells are permissible for entry of prototypic “late” isolates of FIV such as B2542 [44] which are CRD2 independent. Pseudotypes bearing GL8 and B2542 Envs were tested in parallel as representative “early” and “late” pseudotypes, respectively. Each bar represents mean luciferase activity (cpm) ± standard error (*n* = 3). The luciferase activity of pseudotypes on MCC cells expressing the CD134 chimaera containing the human CRD2 domain (FFH; grey bars) determined the subsequent pseudotype classification. All of the pseudotypes bearing SV1 Envs shared similar phenotypes with the pseudotypes bearing the Env of the “early”, CRD2-dependent GL8 strain.

We assessed the receptor utilisation of the twelve autologous Envs isolated from SV1 by using HIV(FIV)-luc pseudotypes. While GL8 Env supported infection of cells expressing feline but not human CD134, the B2542 Env supported infection of cells expressing either feline CD134, or the feline × human CD134 chimaera, expressing the first cysteine rich domain (CRD1) of feline CD134 in the context of human CD134 [42]. In comparison with the GL8 and B2542 Envs, all SV1 Envs were highly dependent on the cysteine-rich domain 2 (CRD2) of CD134 (Fig. 3), displaying a “GL8-like” phenotype similar to that of “early”, acute isolates of FIV that are likely to be transmitted in the field [34,42,43].

## 4 Discussion

Despite several HIV-1 vaccine efficacy studies in human volunteers [9] and the FIV vaccine having been available commercially for 12 years, the mechanisms of vaccine induced protection against lentiviral infection have not been examined in the field. Experimentally, the FIV vaccine did not protect cats against heterologous challenge with the virulent primary GL8 isolate [22]. However, since the natural challenge dose in FIV infection remains undefined, the challenge dose used in experimental studies might be too stringent.

We hypothesised that, if vaccinated cats could be identified, we might find evidence of subsequent infection following natural exposure. Given that the FIV vaccine affords 80% protection [43], we predicted that approximately 20% of vaccinated cats exposed to FIV would become infected. Here, we identified one cat, SV1, which had been vaccinated and tested provirus positive. SV1 had been vaccinated against FIV annually for at least three years, with the last vaccination administered three months prior to FIV diagnosis and death.

Phylogenetic analysis of full length *env* sequences revealed that cat SV1 was infected with a recombinant clade A/B isolate of FIV, the major parent being related to the Clade A isolate GL8. Furthermore, the receptor utilisation phenotype of the SV1 Env variants resembled that of GL8, characteristic of the phenotype displayed by “early” isolates circulating during the acute phase of infection and requiring the CRD2 domain of CD134 for infection [44,45]. Hence it is likely that the Env variants isolated from SV1 had been transmitted recently, raising the question: were the immune responses induced by FIV vaccination insufficient to protect cat SV1 against infection with a recombinant virus displaying the “acute” phenotype? The incomplete medical history of this case prevents a definitive conclusion, since the vaccination and FIV status of cat SV1 prior to 2006 was not documented and so it is possible that the cat could have been infected prior to vaccination. The incomplete medical history of cat SV1 highlights the challenges faced in assessing vaccine efficacy in the field; the compliance of owners and veterinarians in providing detailed clinical histories and in following the recommendations of the vaccine manufacturer cannot be assumed in real life situations. It is the responsibility of veterinarians to provide information to owners pertaining to the risks and benefits of vaccination and to emphasise that cats should always be tested for FIV infection prior to vaccination.

Using a rare panel of plasma samples from Australian cats vaccinated against FIV, we assessed the breadth and potency of NAbs induced by vaccination. None of the plasma samples displayed broad cross-reactivity against a panel of pseudotypes bearing Envs from either reference or field isolates. Only 50% of the vaccinated cats strongly neutralised the pseudotype bearing KKS Env, the sequence of which closely resembles that of FIV-Petaluma [33], one component of the divalent FIV vaccine [21]. A strong NAb response had been proposed as a correlate of protection [46,47] and a crucial component of humoral immunity against virus infections [48,49]. Initial studies reported that NAbs recognising the homologous Petaluma and Shizuoka strains were detected in most vaccinated cats and eight of twelve vaccinated cats neutralised the heterologous FIV Bangston isolate, leading to the conclusion that the two isolates of FIV within the vaccine might act synergistically to enhance the development of NAbs against heterologous strains of FIV [2]. However, another study suggested that vaccine induced NAbs might not be a crucial component of FIV vaccine induced immunity [50], because strong NAb responses were elicited in only three of ten vaccinated cats.

It is possible that plasma SV1 strongly neutralised all of the autologous pseudotypes as a result of antigenic stimulation following FIV infection. It was demonstrated experimentally that, following challenge with the virulent primary GL8 isolate, viral loads in vaccinated cats were significantly enhanced compared to non-vaccinated controls [22]. Given the onset of severe clinical signs in SV1, it could be speculated that vaccination had led to enhanced infection, followed by antigenic hyper-stimulation and hence a robust autologous NAb response might have resulted from the immune system having been primed by vaccination. Insufficient sample was available to assess the plasma viral loads in cat SV1 to test this hypothesis.

It is unclear whether sterilising immunity following FIV vaccination can be achieved in the absence of broadly cross-reactive NAbs. Mechanisms of blocking retroviral infection other than direct neutralisation of free viral particles, including antibody dependent cell mediated cytotoxicity (ADCC), antibody dependent cell mediated viral inhibition (ADCVI) [51] and cellular immunity, which also play roles in controlling retroviral infections were not studied here. Although cellular immunity is important for controlling retroviral infections [52], HIV vaccine candidates eliciting cellular immunity have been ineffective [53–55]. However, FIV vaccination elicits strong adaptive T cell immunity [21], protecting cats against homologous challenge in the absence of NAbs [56].

The present study highlights the need for rigorous evaluation of the FIV vaccine and the challenges associated with such studies under field conditions. The small number of cats tested and the lack of detailed clinical histories for some of them limit the conclusions that can be drawn from the study and demonstrate that a larger number of subjects will be required to comprehensively assess vaccine efficacy in the field.

## 5 Conclusions

This study demonstrated that FIV vaccination induces NAbs against one of the vaccine strains in the majority of vaccinates, potentially a useful marker to identify cats likely to be protected following challenge. Since immune correlates of protection against FIV vaccination remain incompletely understood and recombinant strains of FIV in the field are abundant [57], further studies are warranted to fully assess FIV vaccine efficacy under field conditions. Given the failure of phase III HIV vaccine clinical trials [10–15] and the increased risk of HIV acquisition in some cases [16], are further trials on human volunteers justified before the mechanisms of protection induced by FIV vaccination have been identified? It is apparent that differences between FIV and HIV-1 are more profound than those between HIV-1 and its simian counterpart. Paradoxically, these differences, and the fact that FIV has coexisted with its natural host for longer than HIV-1, may prove crucial to better understanding the interplay between lentiviruses and their hosts and to developing an effective human lentiviral vaccine.

## Contributors

Conceived and designed the experiments: PB, JAB, BJW, MJH; Performed the experiments and analysed the data: PB, NT, MH; Wrote the manuscript: PB, JAB, NT, BJW, MJH.

## Conflict of interest statement

The authors declare that they have no conflict of interest.

## Figures and Tables

**Table 1:** Clinical history of Fel-O-Vax FIV vaccinated cats. Age is accurate for the blood sample collection dates. All cats were tested both by FIV ELISA and FIV PCR, with the exception of two cats kept entirely indoors, which were tested by FIV PCR. One vaccinated cat, SV1 tested positive for FIV proviral DNA, while all others were negative. Breed: DSH—domestic short hair, DMH—domestic medium hair, DLH—domestic long hair. Sex: F—female, FS—female spayed, MN—male neutered. Housing: I—indoor, O—outdoor. y—years. n/a—not available.

Cat	Breed	Sex	Age (y)	Housing	ELISA	PCR	Date of PCR testing	Date of FIV vaccination	Blood collection	Chief complaint	Diagnosis	Other
SV1	DSH	MN	11	I/O	+	+	15.12.09	Oct 2006, Nov 2007, Oct 2008, Sep 2009	09.12.09	No nasal and facial sensation, drooling saliva	Trigeminal nerve paralysis, cavernous sinus syndrome	Renomegaly, fights, unknown FIV status at the first vaccination
SV2	DMH	FS	10	I/O	+	−	26.11.08	Oct 2008	19.11.08	Lethargy and inappetence	Anaemia, hepatitis, splenomegaly, inflammatory bone marrow disease	–
SV3	Ragdoll	FS	3	I	+	−	02.11.09	Regularly since kitten	06.05.10	Lethargy, inappetence and weight loss	Idiopathic hypercalcemia	Renal insufficiency
SV4	Burmese	MN	6	I/O	+	−	13.05.09	Unknown	25.06.09	Lethargy, depression, weight loss, multiple joint effusion	Polyarthritis, immune mediated (?)	Fight wounds
SV5	Abyssinian	MN	10	I/O	+	−	15.07.09	Regularly, last one Jan 2009	23.07.09	Sneezing, ocular and nasal discharge	Active chronic rhinitis	–
SV6	DSH	FS	9	I	+	−	16.10.09	Unknown	14.10.09	Sudden onset ataxia	Meningioma, right occipital lobe	–
SV7	DLH	MN	12	I/O	+	−	09.03.10	Unknown	02.03.10	Presented for radio-iodine treatment	Hyperthyroidism	–
SV8	DSH	MN	6	I/O	+	−	Unknown	Unknown	26.10.10	Swollen left hind leg	Non-regenerative anaemia	Fight wounds
SV9	DSH	FS	8	I	n/a	−	15.12.14	Regularly since kitten	30.04.09	Acute vomiting	Pancreatitis	–
SV10	British short hair	F	1	I	n/a	−	15.12.14	Once as a kitten	20.07.09	Pyrexia, lethargy, inappetence	Effusive feline infectious peritonitis	Euthanised following diagnosis

**Table 2:** Classification of neutralisation potency of plasma samples.

	Neutralisation potency				
	Absent	Weak	Moderate	Strong	
Fold neutralisation	1–1.6	1.7–2.4	2.5–5.5	5.6–10	10,000
% Neutralisation	0–39	40–59	60–80	81–90	100

**Table 3:**
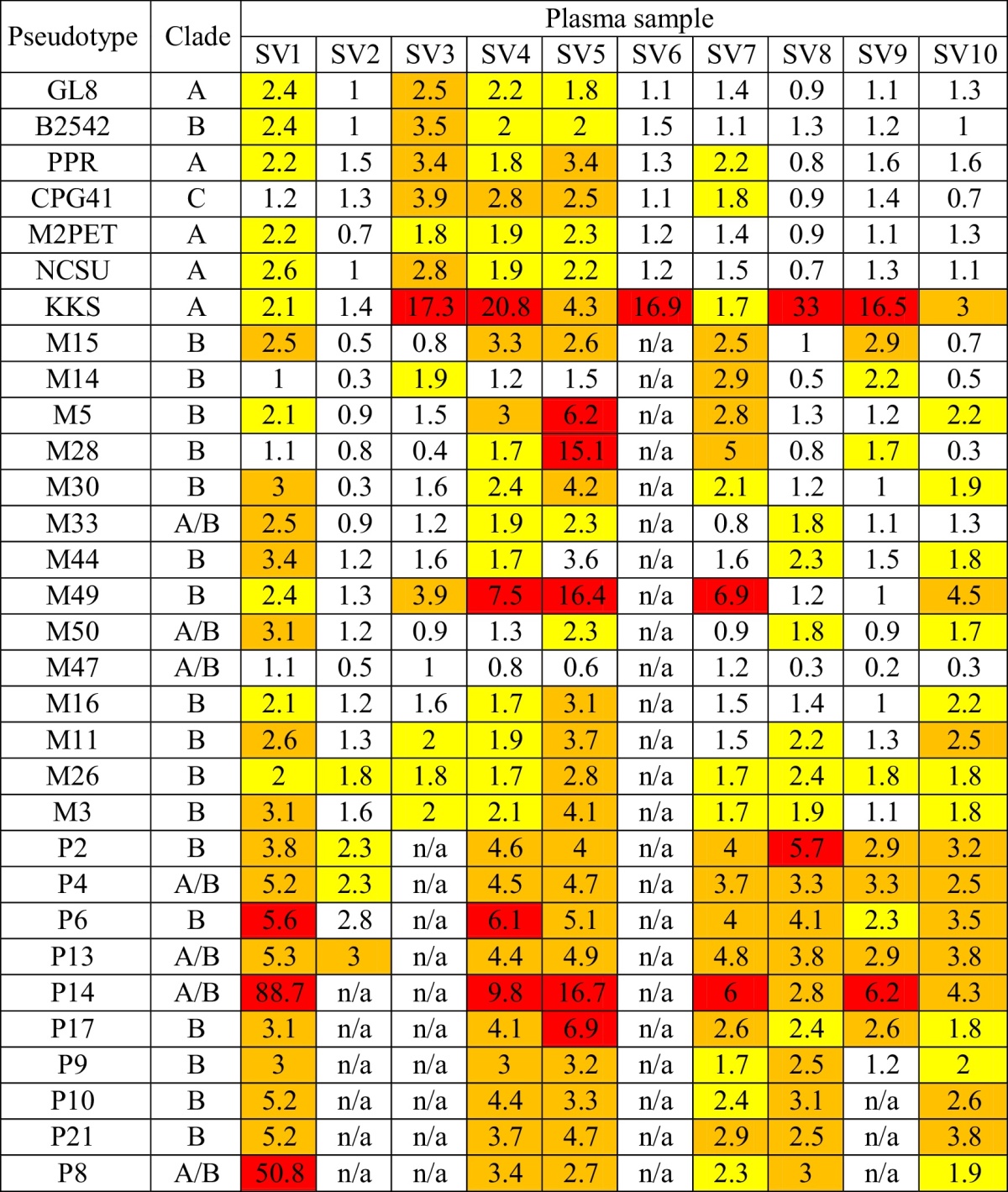
Neutralisation potency of plasma samples from 10 vaccinated cats, expressed as fold neutralisation. Samples were assessed against a panel of pseudotypes bearing 7 reference Envs (GL-8, [27]; B2542, [28]; PPR, [29]; CPG41, [30]; M2PET, [31] NCSU, [32] and KKS, [33]) and 24 wild type Envs isolated from US cats that had been naturally infected with FIV [26]. Phylogenetic classification of the Env clade is included. Weak, moderate or strong neutralisation is indicated in yellow, orange and red, respectively. Sample volumes from cats SV2, SV3, SV6 and SV9 were limited and were insufficient for all analyses. n/a—Not available.

## Appendix A Supplementary data

Figs. S1–S3 and Table S1

Fig. S1 Similarity analysis of SV1 recombinant sequence and two reference sequences (clade A, GL8 [GenBank: X69494] in red and clade B, USIL2489 [GenBank: U11820.1] in green) performed in SimPlot. Each line represents the percentage similarity of the query SV1 sequence to the reference sequences. Recombination breakpoints are located at the intersections of the red and green curves.

Fig. S2 Highlighter diagram showing locations of nucleotide substitutions in each of 24 *env* sequences from cat SV1. The positions of mutations within each sequence in comparison to reference F1 *env* are indicated on the *X*-axis. Nucleotide substitutions are colour coded according to the legend on *Y*-axis. Note that *envs* from cat SV1 displayed high homology.

Fig. S3 Maximum likelihood FIV *env* phylogeny based on the HKY model rooted on the reference clade C. The tree is drawn to scale with branch lengths denoting the number of substitutions per site. The tree comprises: one sequence representative of 24 *env* genes from cat SV1 (red node), 43 complete *env* sequences from Memphis and Chicago FIV naturally infected cats [26], 17 full length *env* sequences derived from GenBank; accession numbers: Aomori 1 [D37816], Aomori 2 [D37817.1], FIV C [AF474246.1], Dixon [L00608.1], Dutch [X60725], Fukuoka [D37815.1], Sendai 1 [D37813.1], Shizuoka [D37811.1], UK2 [X69494.1], UK8 [X69496.1], USIL2489 [U11820.1], Yokohama [D37812.1], Petaluma [M25381.1], PPR [M36968.1], Leviano [FJ374696.1], Bangston [AY620002.1], FC1 [AY621093.1], three V3–V5 region sequences representing Clade E: LP3 [D84496], LP20 [D84498], LP24 [D84500] and one shorter 504 bp in length RUS14 [EF447297] sequence. The selected sequences represented a good geographical, intra- and inter-clade distribution, with overall *env* diversity exceeding 26%. Note that sequence SV1 clusters closely with GL8. Vaccine strains: Petaluma (FIV Clade A) and Shizuoka (FIV Clade D) are highlighted in green. Challenge strains (FC1 and Bangston) used in vaccine efficacy trials are highlighted in turquoise.

Table S1 Primers.
