# Supplementary material for: Neutralising antibody response in domestic cats immunised with a commercial feline immunodeficiency virus (FIV) vaccine
Source: Vaccine. 2015 Feb 18;33(8):977–84. doi: 10.1016/j.vaccine.2015.01.028 (PMC4327927; doi:10.1016/j.vaccine.2015.01.028)
Supplement: Fig. S1 — Similarity analysis of SV1 recombinant sequence and two reference sequences (clade A, GL8 [GenBank: X69494] in red and clade B, USIL2489 [GenBank: U11820.1] in green) performed in SimPlot. Each line represents the percentage similarity of the query SV1 sequence to the reference sequences. Recombination breakpoints are located at the intersections of the red and green curves. [file mmc1.docx]

| **Primer** | **Sequence (5'-3')** | **Purpose** |
| --- | --- | --- |
| 2F2 | TATTATTGGCARTTGCAATCTACMTTATC | Blood direct PCR forward primer |
| 1R4 | CCAATAMTCWTCCCAGTCCACCCTT | Blood direct PCR reverse primer |
| SV1Sal1F | GGGTCGACACCATGCTTAGCGATGATTGCAGCC | Second round forward primer |
| SV1Not1R | GGGCGGCCGCCATCATATCTCCTCTTTTTCAGAC | Second round reverse primer |
| VR1012F | CTTTTCTGCAGTCACCGTCG | Sequencing forward primer |
| VR1012R | CTGGATCCAGGCGCCTGGTCTA | Sequencing reverse primer |
| Memphis780F | TGGAATGAGACTATAACAGG | Sequencing forward primer |
| Memphis2090R | GATTACATCCTAATTCTTGCATAG | Sequencing reverse primer |
