# Supplementary figures and images for: Neutralising antibody response in domestic cats immunised with a commercial feline immunodeficiency virus (FIV) vaccine
Source: Vaccine. 2015 Feb 18;33(8):977–84. doi: 10.1016/j.vaccine.2015.01.028 (PMC4327927; doi:10.1016/j.vaccine.2015.01.028)

**
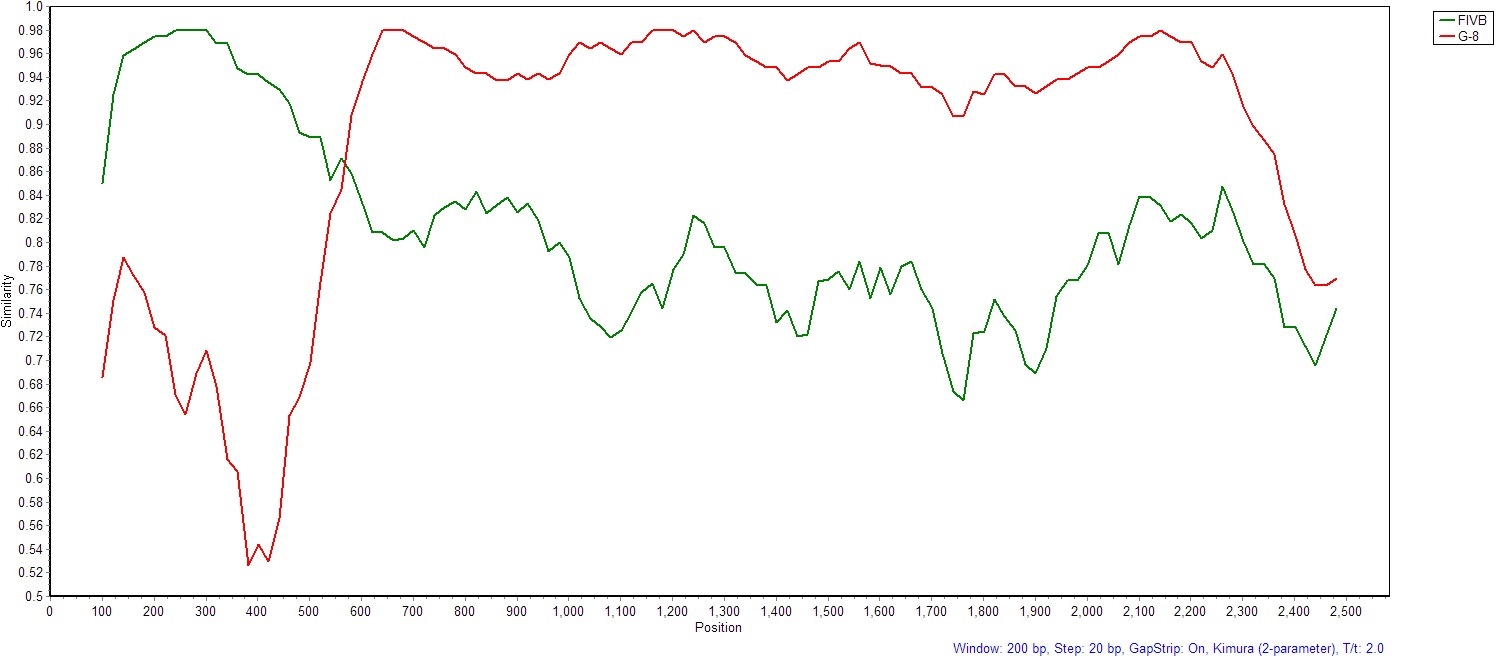
**

Supplement: Fig. S2 — Highlighter diagram showing locations of nucleotide substitutions in each of 24 env sequences from cat SV1. The positions of mutations within each sequence in comparison to reference F1 env are indicated on the X-axis. Nucleotide substitutions are colour coded according to the legend on Y-axis. Note that envs from cat SV1 displayed high homology. [file mmc2.docx]

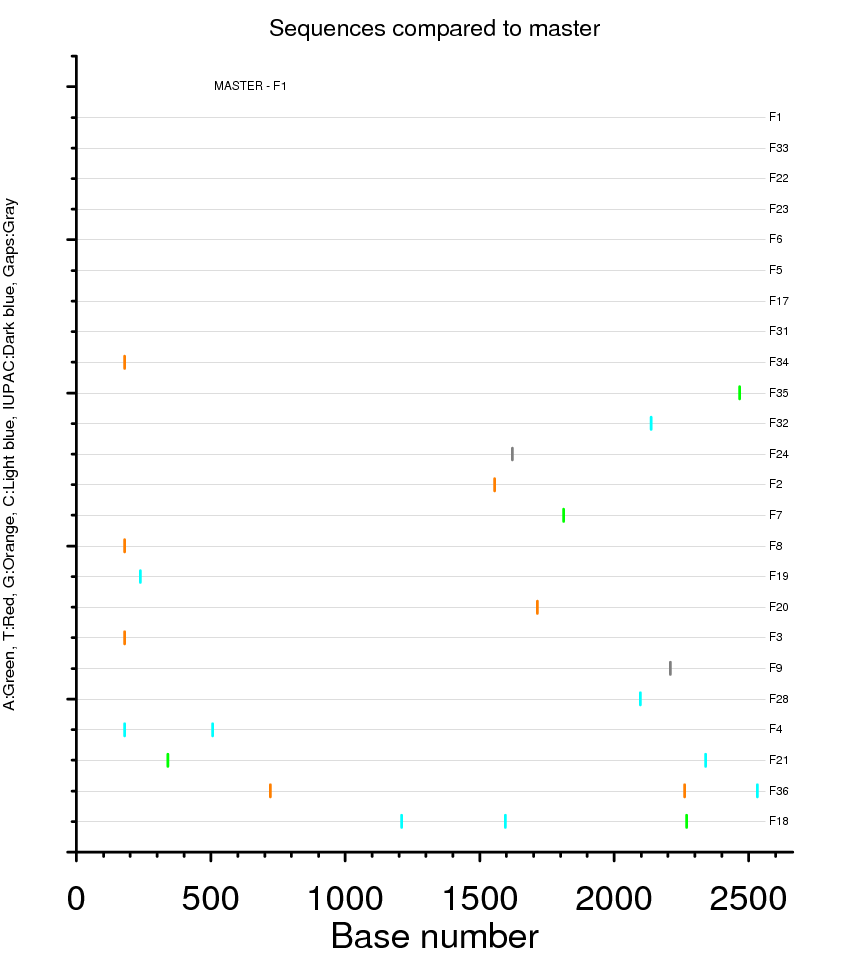

Supplement: Fig. S3 — Maximum likelihood FIV env phylogeny based on the HKY model rooted on the reference clade C. The tree is drawn to scale with branch lengths denoting the number of substitutions per site. The tree comprises: one sequence representative of 24 env genes from cat SV1 (red node), 43 complete env sequences from Memphis and Chicago FIV naturally infected cats [26], 17 full length env sequences derived from GenBank; accession numbers: Aomori 1 [D37816], Aomori 2 [D37817.1], FIV C [AF474246.1], Dixon [L00608.1], Dutch [X60725], Fukuoka [D37815.1], Sendai 1 [D37813.1], Shizuoka [D37811.1], UK2 [X69494.1], UK8 [X69496.1], USIL2489 [U11820.1], Yokohama [D37812.1], Petaluma [M25381.1], PPR [M36968.1], Leviano [FJ374696.1], Bangston [AY620002.1], FC1 [AY621093.1], three V3–V5 region sequences representing Clade E: LP3 [D84496], LP20 [D84498], LP24 [D84500] and one shorter 504 bp in length RUS14 [EF447297] sequence. The selected sequences represented a good geographical, intra- and inter-clade distribution, with overall env diversity exceeding 26%. Note that sequence SV1 clusters closely with GL8. Vaccine strains: Petaluma (FIV Clade A) and Shizuoka (FIV Clade D) are highlighted in green. Challenge strains (FC1 and Bangston) used in vaccine efficacy trials are highlighted in turquoise. [file mmc3.docx]

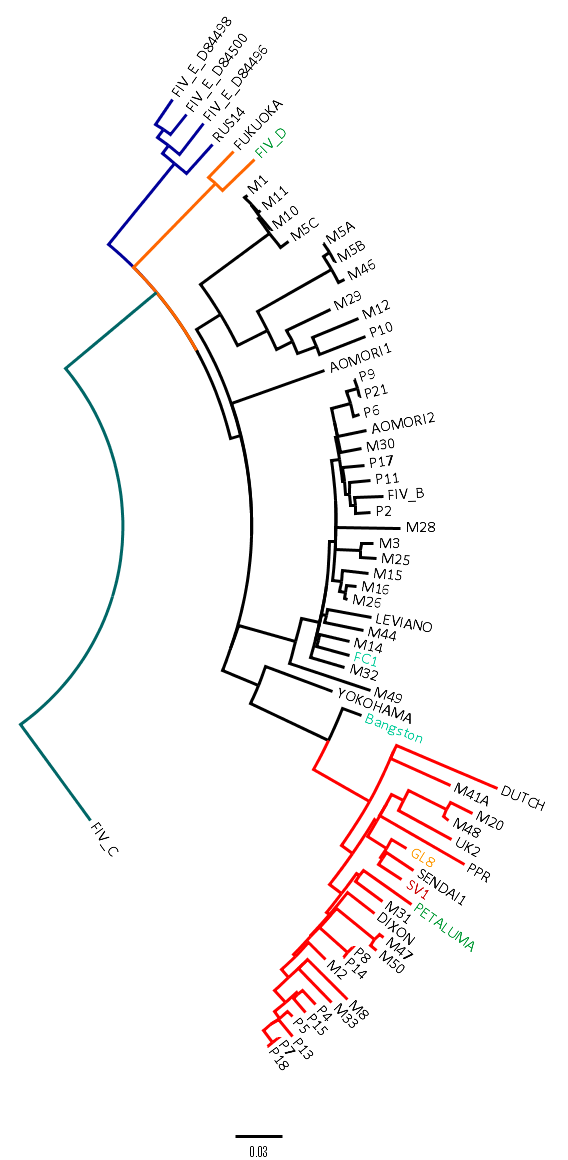

Supplement: Table S1 — Primers. [file mmc4.docx]
